# Acinetobacter baumannii GC2 Sublineage Carrying the *aac*(*6*′)-*Im* Amikacin, Netilmicin, and Tobramycin Resistance Gene Cassette

**DOI:** 10.1128/spectrum.01204-23

**Published:** 2023-07-06

**Authors:** Christopher J. Harmer, Steven J. Nigro, Ruth M. Hall

**Affiliations:** a School of Life and Environmental Sciences, The University of Sydney, New South Wales, Australia; University at Albany

**Keywords:** *Acinetobacter baumannii* evolution, global clone 1, capsule locus, aminoglycoside resistance, insertion sequence ISAba1, antibiotic resistance, genomic island, insertion sequence, *aac(6′)-Im*

## Abstract

The aminoglycoside antibiotics amikacin, gentamicin, and tobramycin are important therapeutic options for Acinetobacter iinfections. Several genes that confer resistance to one or more of these antibiotics are prevalent in the globally distributed resistant clones of Acinetobacter baumannii, but the *aac*(*6*′)-*Im* (*aacA16*) gene (amikacin, netilmicin, and tobramycin resistance), first reported in isolates from South Korea, has rarely been reported since. In this study, GC2 isolates (1999 to 2002) from Brisbane, Australia, carrying *aac*(*6*′)-*Im* and belonging to the ST2:ST423:KL6:OCL1 type were identified and sequenced. The *aac*(*6*′)-*Im* gene and surrounds have been incorporated into one end of the IS*26*-bounded AbGRI2 antibiotic resistance island and are accompanied by a characteristic 70.3-kbp deletion of adjacent chromosome. The compete genome of the 1999 isolate F46 (RBH46) includes only two copies of ISAba1 (in AbGRI1-3 and upstream of *ampC*) but later isolates, which differ from one another by <10 single nucleotide differences (SND), carry two to seven additional shared copies. Several complete GC2 genomes with *aac*(*6*′)-*Im* in an AbGRI2 island (2004 to 2017; several countries) found in GenBank and two additional Australian A. baumannii isolates (2006) carry different gene sets, KL2, KL9, KL40, or KL52, at the capsule locus. These genomes include ISAba1 copies in a different set of shared locations. The distribution of SND between F46 and AYP-A2, a 2013 ST2:ST208:KL2:OCL1 isolate from Victoria, Australia, revealed that a 640-kbp segment that includes KL2 and the AbGRI1 resistance island replaces the corresponding region in F46. Over 1,000 A. baumannii draft genomes also include *aac*(*6*′)-*Im*, indicating that it is currently globally disseminated and significantly underreported.

**IMPORTANCE** Aminoglycosides are important therapeutic options for treatment of Acinetobacter infections. Here, we show that a little-known aminoglycoside resistance gene, *aac(6′)-Im* (*aacA16*), that confers amikacin, netilmicin, and tobramycin resistance has been circulating undetected for many years in a sublineage of A. baumannii global clone 2 (GC2), generally with a second aminoglycoside resistance gene, *aacC1*, which confers resistance to gentamicin. These two genes are commonly found together in GC2 complete and draft genomes and globally distributed. One isolate appears to be ancestral, as its genome contains few ISAba1 copies, providing insight into the original source of this insertion sequence (IS), which is abundant in most GC2 isolates. Tracking ISAba1 spread can provide a simple means to track the development and ongoing evolution as well as the dissemination of specific lineages and detect the formation of many sublineages. The complete ancestral genome will provide an essential base point for tracking this process.

## INTRODUCTION

Carbapenems have for some time been the first-choice antibiotics for treating Acinetobacter baumannii infections, but with the global spread of carbapenem-resistant A. baumannii, clinicians have turned to other classes of antibiotics, including the aminoglycosides (1, 2). However, resistance to aminoglycoside antibiotics used for the treatment of A. baumannii infections, gentamicin, tobramycin, and amikacin, is also common and represents a major problem in the treatment of infections across the world (3–6). Many A. baumannii that have developed resistance to aminoglycosides are now classified as extensively resistant or pan-resistant. To date, the majority of A. baumannii isolates that are extensively antibiotic resistant belong to two globally disseminated clones, global clone 1 (GC1) and global clone 2 (GC2).

The most common mechanism of aminoglycoside resistance in A. baumannii involves the production of aminoglycoside-modifying enzymes and ribosomal protection via methylation (5, 7). The *aacC1* gentamicin resistance gene is widespread, as it is found in a gene cassette in a class 1 integron in genomic resistance islands in both GC1 (8, 9) and GC2 (3) A. baumannii. Under normal circumstances, the *aphA1* gene, which is found in the same resistance islands, confers resistance to only kanamycin and neomycin, but it can also confer tobramycin resistance when it is significantly amplified (6, 10). The *armA* gene, found in the AbGRI3 island of many recent GC2 A. baumannii isolates (11), confers resistance to all clinically relevant aminoglycosides (gentamicin, kanamycin, amikacin, tobramycin, and netilmicin) (12). AbGRI3 also generally includes the *aacA4* gene cassette. The *aadB* gentamicin and tobramycin resistance gene cassette is usually associated with a small plasmid, pRAY* (13), and *aphA6* (amikacin resistance) is found in transposon Tn*aphA6* (14) in plasmids or in the chromosome. The cassette-associated *aac*(*6*′)-*Im* gene, which confers resistance to amikacin, netilmicin, and tobramycin, has been found in A. baumannii isolates from an outbreak in South Korea (15), but it has not often been reported since and its context remains unknown.

The *aac*(*6*′)-*Im* gene was originally found in a gene cassette associated with a class 1 integron in a Citrobacter freundii from Belgium and called *aac*(*6*′)-*Il* (16), but the designation *-Il* was not available and the name was later corrected to *aac*(*6*′)-*Im* by the authors (17). This gene has also been referred to as *aac*(*6*′)-*Ip* without explanation for the name change (18). It has also been called *aacA16* (19). To be consistent with the literature, here we use the *aac*(*6*′)-*Im* designation.

A set of 33 isolates selected from a collection of 483 multiresistant A. baumannii isolates recovered at the Royal Brisbane and Women’s Hospital, mainly during outbreaks in 2002 and 2006, were typed using the Oxford multilocus sequence typing (MLST) scheme, and two main groups were identified as sequence type 92 (ST92) and ST69 (20). However, the identity of most of the resistance genes present was not examined. These Oxford sequence type (ST^Ox^) isolates were formerly ST22 and ST28, respectively (see Table 3 in reference 20) but are now known to in fact be ST208 and ST423 (see reference 21 for details). ST208 (ST92) was the dominant lineage in this subset (20), and we have previously reported on aminoglycoside resistance genes found in some ST208 isolates that were part of a different subset (22) of the same Brisbane collection (3). The ST423 (ST69) isolates had been present in the hospital between 1998 and 1999, prior to a second introduction of an ST423 from Indonesia in 2002 that preceded an outbreak (20).

In this study, we have examined four ST423 A. baumannii isolates from Brisbane that we found contained the *aac*(*6*′)-*Im* gene using PCR. One of these isolates, F44, had been shown to belong to ST423 (21). Another ST423 isolate, F4 (also known as RBH4), had previously been used to determine the KL6 capsular polysaccharide structure (23). Whole-genome sequences (WGS) were determined and regions that include antibiotic resistance genes were assembled and analyzed. The locations of ISAba1 copies in the chromosome and single nucleotide difference (SND) analysis were used to examine their relatedness to one another and to further isolates with the *aac*(*6*′)-*Im* resistance gene found in our collection of draft genomes of Australian GC2 isolates. Complete genomes containing the *aac*(*6*′)-*Im* gene cassette found in the GenBank nucleotide database were also examined to track the evolution of the resistance island containing *aac*(*6*′)-*Im* and of the GC2 lineages that carry it. Finally, the prevalence and global distribution of isolates carrying the *aac*(*6*′)-*Im* gene cassette were examined by interrogating the A. baumannii draft genomes in the WGS database.

## RESULTS

### GC2 isolates containing *aac*(*6*′)-*Im*.

Initial screening using PCR had shown that four isolates, F4, F44, F46, and F48, from our small collection of multiply antibiotic-resistant isolates recovered in Brisbane between 1999 and 2002 belong to GC2 and carry the *aac*(*6*′)-*Im* (*aacA16*) resistance gene, explaining an unusual combination of resistance to clinically relevant aminoglycoside antibiotics, namely, amikacin, netilmicin, and tobramycin. Additional resistance to gentamicin is supplied by the *aacC1* gene. All isolates were also resistant to aminoglycosides, streptomycin, spectinomycin, and kanamycin, with resistance to neomycin variable. They were also resistant to ampicillin, ceftazidime, cefotaxime, sulfamethoxazole, tetracycline, trimethoprim, and the quinolones ciprofloxacin and nalidixic acid but were susceptible to the carbapenems imipenem and meropenem.

The draft genomes of these isolates were determined and their relationships examined (Table 1). For simplicity, the strain profiles include the sequence type (ST) in the Institut Pasteur (ST^IP^) and the Oxford (ST^Ox^) MLST schemes together with the identity of the gene clusters at the capsule (KL) and lipopolysaccharide outer core (OCL1) loci. Profiles are designated ST^IP^:ST^OX^:KL:OCL (24). F4, F44, F46, and F48 were distinct from the previously described GC2 (ST2:ST208:KL2:OCL1) members of the Brisbane collection (3, 14) and formed a distinct GC2 group with the profile ST2:ST423:KL6:OCL1. Hence, they correspond to the ST69 group identified by Runnegar et al. (20). The *oxaAb* gene in the four isolates encodes the OXA-66 variant, as expected for GC2 isolates.

**TABLE 1:** Australian ST2:ST423:KL6:OCL1 genomes containing *aac(6′)-Im*

Isolate	Yr	*ampC* ^*a*^	Resistance genes	Accession no.
F46	1999	19	AbGRI1-3: *strA*, *strB*, *sul2*, *tetA*(B)	CP096575
			AbGRI2: *aac(6′)-Im*, *aacC1*, *aadA1*,^*b*^ *aphA1b*, *bla*_TEM-1D_, *sul1*	
F4	2002	19	AbGRI1-3: *strA*, *strB*, *sul2*, *tetA*(B)	JAKZLG
			AbGRI2: *aac(6′)-Im*, *aacC1*, *aadA1*,^*b*^ *bla*_TEM-1D_, *sul1*	
F44	2002	19	AbGRI1-3: *strA*, *strB*, *sul2*, *tetA*(B)	JAQJIR
			AbGRI2: *aac(6′)-Im*, *aacC1*, *aadA1*,^*b*^ *bla*_TEM-1D_, *sul1*	
F48	Unknown^*c*^	19	AbGRI1-3: *strA*, *strB*, *sul2*, *tetA*(B)	JAQJIQ
			AbGRI2: *aac(6′)-Im*, *aacC1*, *aadA1*,^*b*^ *aphA1b*, *bla*_TEM-1D_, *sul1*	
K17	2002	19	AbGRI1-3: *strA*, *strB*, *sul2*, *tetA*(B)	JAQJIP
			AbGRI2: *aac(6′)-Im*, *aacC1*, *aadA1*,^*b*^ *bla*_TEM-1D_, *sul1*	

a *ampC-19* differs from *ampC-2* by two SNDs.

b Split by an IS*26*-mediated inversion.

c Date not recorded, but the isolate was collected between 1999 and 2002 (22).

### Complete genome of F46.

To facilitate the examination of resistance islands and other features in the group, the complete genome of strain F46, recovered in 1999, was determined. F46 is the oldest strain in this group of *aac*(*6*′)-*Im* strains and is among the earliest GC2 A. baumannii isolates recovered in Australian hospitals. The Unicycler hybrid assembly for strain F46 produced a 3,878,974-bp circular chromosome (GenBank accession number CP096575) and no plasmids.

A copy of ISAba1 in the appropriate orientation to enhance expression was found in the usual position (25) 9 bp upstream of the chromosomal *ampC* gene (allele 19), and this accounts for the resistance to ceftazidime and cefotaxime. An S81L substitution in GyrA together with an S84L substitution in ParC accounts for the nalidixic acid and ciprofloxacin resistance.

**FIG 1:**
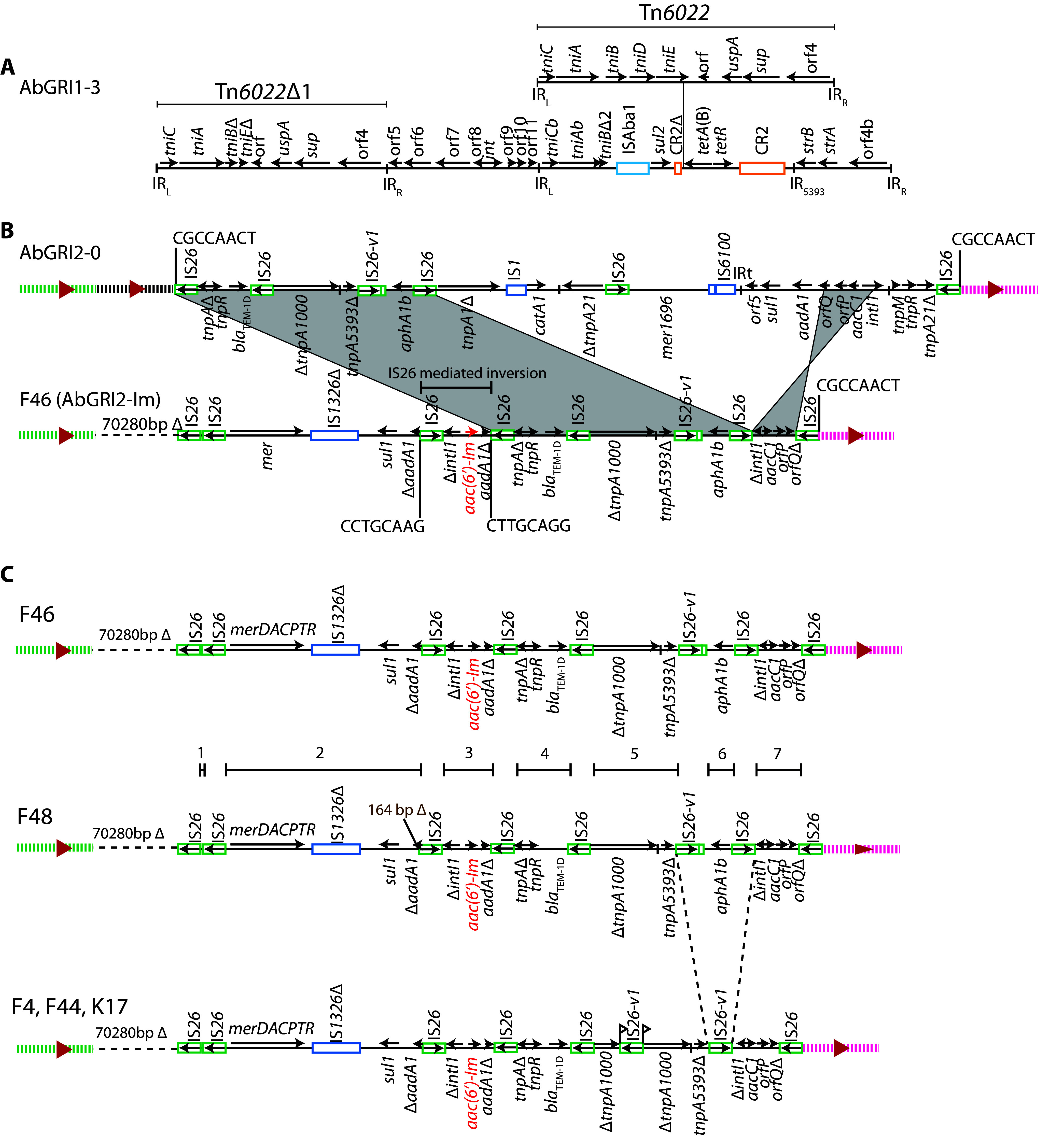
Antibiotic resistance islands in ST2:ST423:KL6:OCL1 Australian A. baumannii. (A) AbGRI1-3 island in F46, F4, F44, F48, and K17. (B) AbGRI2-Im in F46 compared to the AbGRI2-0 progenitor. (C) AbGRI2-Im variants in F4, F44, F48, and K17. Horizontal arrows show the extent and direction of reading frames, with names above and below. Complete and partial copies of insertion sequences and CR2 are shown as colored boxes, with names above. For IS*26*, an internal arrow indicates the IS orientation. Shading indicates shared regions, and the backbone adjacent to AbGRI2-Im is indicated by green or pink dashed lines. Dashed horizontal lines indicate the backbone deletion, with the size of the deletion shown above. Target site duplications (TSDs) are shown as 8-base sequences above vertical lines. The internal contigs assembled are indicated above the F48 line. Illustrations are drawn to scale from GenBank accession numbers CP096575 (F46), JAKZLG000000000 (F4), JAQJIR000000000 (F44), JAQJIQ000000000 (F48), and JAQJIP000000000 (K17). AbGRI2-0 is drawn from the reconstructed A320 sequence (GenBank accession number CP032055).

The acquired antibiotic resistance genes in the F46 genome (Table 1) were distributed between the two antibiotic resistance islands, AbGRI1 and AbGRI2, usually found in GC2 isolates. A previously described variant of AbGRI1 was present in F46. This variant, originally found in the genome of MDR-TJ (26) but now known as AbGRI1-3 (27), has a complex transposon structure (Fig. 1A). It interrupts the chromosomal *comM* gene and is surrounded by a 5-bp target site duplication (TSD). AbGRI1-3 contains the *sul2* (sulfonamide), *tetA*(B) (tetracycline), and *strA* and *strB* (streptomycin) resistance genes (Fig. 1A). This 38,810-bp island differs from the proposed progenitor AbGRI1-0 (28) by a 2.85-kbp deletion in Tn*6022*, the addition of a 2.58-kbp fragment that includes *tetA*(B), and the incorporation of an additional copy of Tn*6022* at the end of the *tetA*(B) gene.

The remaining acquired antibiotic resistance genes in F46, *aacC1*, *aac*(*6*′)-*Im* (*aacA16*), *aphA1b*, and *bla*_TEM-1D_, were found in a version of AbGRI2, here called AbGRI2-Im (Fig. 1B), that has undergone multiple IS*26*-mediated rearrangements relative to the proposed precursor AbGRI2-0 (29). A 13.5-kbp fragment bounded by directly oriented copies of IS*26* has been incorporated at the left end of the island, and 70.3 kbp of the adjacent chromosome corresponding to bases 1212167 to 1282446, locus tags A320_01150 to A320_01207, in the GC2 reference strain A320 (GenBank accession number CP032055) (29) has been deleted. The inserted fragment contains a partial class 1 integron with the *aac*(*6*′)-*Im* gene cassette and an *aadA1* cassette that has been split by an IS*26*-mediated inversion. The integron is next to a novel mercuric resistance operon (*merDACPTR*) that shares <95% nucleotide identity with *mer* operons characterized in detail to date (30, 31). The *merD* gene has been truncated by an IS*26* and the IR*_mer_* is not present. In addition, multiple IS*26*-mediated deletions have removed the remnants of Tn*9*, Tn*21*, *mer* of Tn*1696*, and part of the class 1 integron that are found in AbGRI2-0.

### Antibiotic resistance islands in the genomes of F4, F44, F48, and K17.

The enhanced draft genome sequences of F4, F44, and F48, which also contained *aac*(*6*′)-*Im*, included 66 to 100 contigs (see Table S1 in the supplemental material). Screening draft genomes of our collection of over 200 Australian isolates for further isolates carrying KL6 revealed an additional ST2:ST423:KL6:OCL1 isolate, K17, carrying the *aac*(*6*′)-*Im* gene that was recovered in Adelaide in 2002. K17 is isolate 6856390 in reference 32, and the enhanced draft genome of K17 is also reported here (Table S1).

The antibiotic resistance phenotypes of F48, F4, F44, and K17 and the resistance genes present were similar to those of F46. Like F46, they all included a copy of ISAba1 located 9 bp upstream of the *ampC* gene (allele 19) conferring resistance to ceftazidime and cefotaxime and the ParC and GyrA substitutions found in F46 accounting for the resistance to nalidixic acid and ciprofloxacin. F44 and F48 also include a copy of ISAba1 located 7 bp upstream of *oxaAb* (OXA-66). ISAba1 is in the correct orientation to drive expression of *oxaAb*, although F48 and F44 were susceptible to imipenem and only displayed reduced susceptibility to meropenem (2 μg/mL relative to 0.36/0.72 for F4 and F46; >8 μg/mL is considered resistant). This is consistent with reports that, even when overexpressed by the outward-facing promoter of ISAba1, OXA-66 does not confer resistance to meropenem and additional mutations are required to achieve this phenotype (33, 34). The enhanced draft assemblies of F4, F44, F48, and K17 each also included AbGRI1-3 (Table 1).

Due to the presence of multiple copies of IS*26*, AbGRI2 was initially in 7 contigs in F48 and 6 contigs in F4, F44, and K17 which all lack the *aphA1b* kanamycin and neomycin resistance gene (Table 1). After assembly using a series of linkage PCRs, AbGRI2 in each of these isolates was found to be similar to AbGRI2-Im in F46 and all of them had an adjacent backbone deletion that was identical to the backbone deletion in F46 (Fig. 1C). In comparison to AbGRI2-Im in F46, F48 includes a 164-bp IS*26*-mediated deletion into *aadA1*. An additional copy of IS*26* interrupting *tnpA1000* was found in F4, F44, and K17, and homologous recombination between the two copies of IS*26* bounding Tn*6020* has removed *aphA1b*.

### Relationships between Australian AbGRI2-Im isolates.

**FIG 2:**
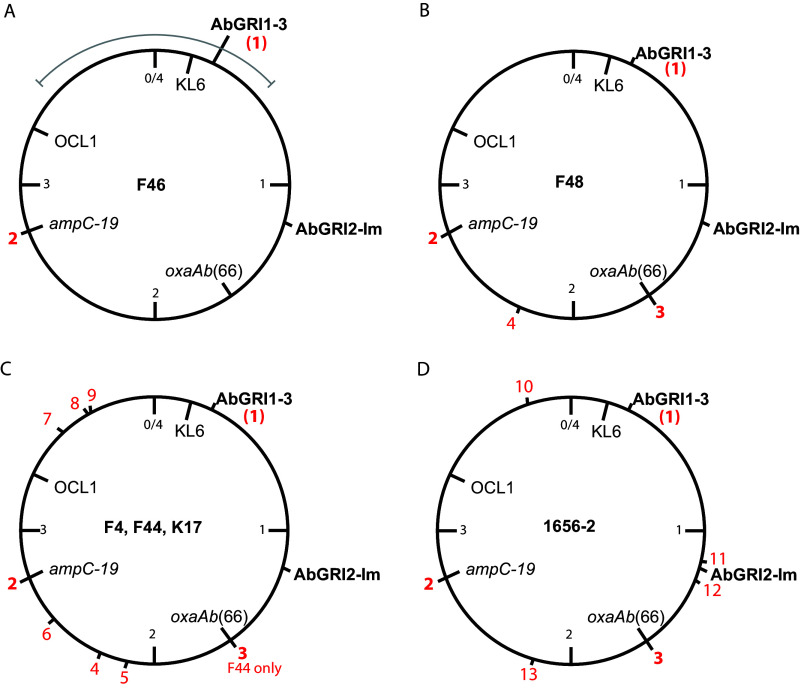
ISAba1 positions in F46 (A), F48 (B), F4, F44, and K17 (C), and the reconstructed 1656-2 (D). The locations of key features AbGRI1, AbGRI2, *oxaAb*, *ampC*, KL, and OCL are marked. ISAba1 positions are indicated by red numbers outside the circle. Bold numbers 1 to 3 indicate ISAba1 upstream of *ampC*, ISAba1 in AbGRI1, and ISAba1 upstream of *oxaAb*, respectively. The gray arc in panel A indicates the position of a recombination patch in AYP-A2.

The relationships were examined further by mapping ISAba1 positions and SND analysis. Only two copies of ISAba1 were detected in the chromosome of the 1999 isolate F46. One is in the AbGRI1-3 resistance region (position 1) and the second is the copy found upstream of the chromosomal *ampC* gene (position 2 [Fig. 2A]). In contrast, we (35) and others (36, 37) have found ISAba1 to be generally abundant in GC2 isolates. As the F46 genome is unusually free of ISAba1 copies, F46 likely represents the ancestor of the related isolates with additional ISAba1 copies in the chromosome.

In addition to ISAba1 copies at positions 1 and 2, F48 (date of isolation unknown) has an ISAba1 upstream of *oxaAb* (position 3) and only one further IS at position 4 in the chromosome (Fig. 2B). The ISAba1 at position 4 is also shared by the remaining isolates, which are all from 2002 and include ISAba1 copies at five further positions (5 to 9) as well as at positions 1 and 2 (Fig. 2C). Thus, it is likely that they evolved from F48. In addition, F44 has the ISAba1 upstream of *oxaAb* (position 3).

Comparison of the number of SNDs between the Australian KL6 isolates further supports the evolutionary links between the isolates. Compared to F46, F48 and each of the later isolates had acquired between 43 and 50 SNDs. Relative to one another, F48 and the 2002 isolates differed from one another only by between 5 and 11 SNDs, supporting a closer relationship. Hence, it is possible that the strain imported from Indonesia in 2002 (20) resembled F48 and that the additional ISAba1 movement events occurred during the outbreak. Patient transfer could explain the strong similarity of the Adelaide isolate.

### The *aac*(*6*′)-*Im* gene in complete A. baumannii genomes from South Korea.

**FIG 3:**
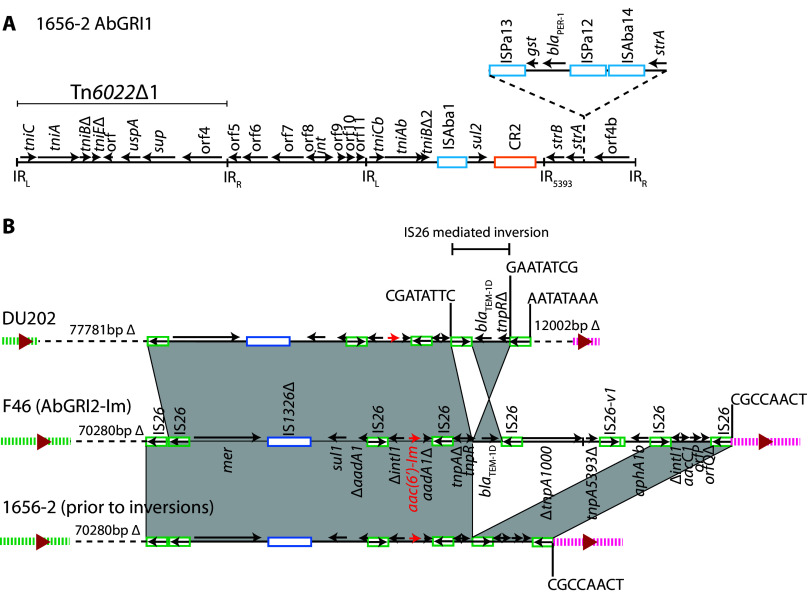
Genomic resistance islands in complete *aac*(*6*′)-*Im*-containing A. baumannii genomes from South Korea. (A and B) AbGRI1 in 1656-2 (A) and AbGRI2-Im (B) variants in DU202 and 1656-2 relative to F46. Horizontal arrows show the extent and direction of reading frames, named only on the F46 configuration. *aac*(*6*′)-*Im* is indicated by a red horizontal arrow. Insertion sequences are shown as boxes. Shading indicates shared regions. The backbone adjacent to AbGRI2-Im is indicated by green or pink dashed vertical lines. Dashed horizontal lines indicate backbone deletions, with the size of the deletion shown above. TSDs are shown as letters above vertical lines. Illustrations are drawn to scale from GenBank accession numbers CP017152 (DU202) and CP001921 (1656-2).

The GenBank nonredundant nucleotide database was queried with the 552-bp *aac*(*6*′)-*Im* gene sequence to determine how widespread the gene was among A. baumannii strains. Identical sequences were found in several complete GC2 genomes (Table 2). However, only two GC2 isolates recovered in South Korea, 1656-2 (38) and DU202 (39), also carried KL6 and shared the same profile (ST2:ST423:KL6:OCL1) as the Australian isolates reported here (Table 2). The genome of 1656-2 (GenBank accession number CP001921) was one of the earliest A. baumannii complete genomes to be reported (38) and was used to define the KL6 capsule locus (40). However, an analysis of the resistance gene content was not reported. 1656-2 carries an unusual configuration of AbGRI1 (Fig. 3A), originally described by Huang et al. (26) and called RI_1656-2_ but here designated AbGRI1_1656-2_. The span of AbGRI1-3 containing the *tetA*(B) gene is missing from the configuration in 1656-2 and an additional fragment containing the *bla*_PER-1_ gene and a second copy of *strA* has been acquired, likely via recombination within *strA*. 1656-2 also includes two plasmids (38).

**TABLE 2:** Complete genomes of GC2/ST2 Acinetobacter baumannii containing *aac(6′)-Im* and the associated novel *mer*

Isolate	Yr	Country	ST^Ox^	*ampC* ^*a*^	KL	Resistance genes	Accession no.
F46	1999	Australia	423	19	6	AbGRI1-3: *strA*, *strB*, *sul2*, *tetA*(B)	CP096575
						AbGRI2: *aac(6′)-Im*, *aacC1*, *aadA1*,^*b*^ *aphA1b*, *bla*_TEM-1D_, *sul1*	
1656-2	<2011	South	423	23	6	AbGRI1-1656-2: *per1*, *strA*, *strB*, *sul2*	CP001921
		Korea				AbGRI2: *aac(6′)-Im*, *aacC1*, *aadA1*,^*b*^ *sul1*	
DU202	Unknown	South	423	23	6	AbGRI1-3: *strA*, *strB*, *sul2*, *tetA*(B)	CP017152
		Korea				AbGRI2: *aac(6′)-Im*, *aadA1*,b *bla*_TEM-1D_, *sul1*	
						AbGRI3: *aphA1*, *armA*, *catB8*, *mph*(E), *msr*(E), *oxa23*, *sul1*	
FDAARGOS_560	Unknown	USA	208	2	2	AbGRI1-5: *strA*, *strB*, *sul2*, *tetA*(B)	CP033862
						AbGRI2: *aac(6′)-Im, aacC1*, *aadA1*,^*b*^ *aphA1*, *bla*_TEM-1D_, *sul1*	
AYP-A2	2013	Australia	208	2	2	AbGRI1-5: *strA*, *strB*, *sul2*, *tetA*(B)	CP024124
						AbGRI2: *aac(6′)-Im*, *aacC1*, *aadA1*,^*b*^ *aphA1*, *bla*_TEM-1D_, *sul1*	
CFSAN093708	2004	USA	208	2	2	AbGRI1-5: *strA*, *strB*, *sul2*, *tetA*(B)	CP061519
						AbGRI2: *aac(6′)-Im*, *aacC1*, *aadA1*,^*b*^ *aphA1*, *bla*_TEM-1D_, *sul1*	
CFSAN093706	2005	USA	208	2	2	AbGRI1-5: *strA*, *strB*, *sul2*, *tetA*(B)	CP061523
						AbGRI2: *aac(6′)-Im*, *aacC1*, *aadA1*,^*b*^ *aphA1*, *bla*_TEM-1D_, *sul1*	
CFSAN093707	2004	USA	208	2	2	AbGRI1-5: *strA*, *strB*, *sul2*, *tetA*(B)	CP061521
						AbGRI2: *aac(6′)-Im*, *aacC1*, *aadA1*,^*b*^ *aphA1*, *bla*_TEM-1D_, *sul1*	
CFSAN093710	2005	USA	208	2	2	AbGRI1-5: *strA*, *strB*, *sul2*, *tetA*(B)	CP061514
						AbGRI2: *aac(6′)-Im*, *aacC1*, *aadA1*,^*b*^ *aphA1*, *bla*_TEM-1D_, *sul1*	
CFSAN093709	2007	USA	208	2	2	AbGRI1-5: *strA*, *strB*, *sul2*, *tetA*(B)	CP061517
						AbGRI2: *aac(6′)-Im*, *aacC1*, *aadA1*,^*b*^ *aphA1*, *bla*_TEM-1D_, *sul1*	
CFSAN093705	2009	USA	208	2	2	AbGRI1-5: *strA*, *strB*, *sul2*, *tetA*(B)	CP061525
						AbGRI2*: aac(6′)-Im*, *aadA1*,^*b*^ *bla*_TEM-1D_, *sul1*	
						Other: *aadB*, *catB3*, *dfrA19*, *mph*(E), *msr*(E), *sul1*	
AB194-VUB	Unknown	Belgium	425	2	40	AbGRI1-5: *strA*, *strB*, *sul2*, *tetA*(B)	CP091351
						AbGRI2: *aac(6′)-Im*, *aadA1*,^*b*^ *sul1*	
AB3-VUB	Unknown	Belgium	448	63	40	AbGRI1-1: *strA*, *strB*, *tetA*(B)	CP091378
						AbGRI2: *aac(6′)-Im*, *aacC1*, *aadA1*,^*b*^ *bla*_TEM-1D_, *sul1*	
CUVET-MIC596	2017	Thailand	1962	2	52	AbGRI1-1: *strA*, *strB*, *tetA*(B)	CP041148
						AbGRI2: *aac(6′)-Im*, *aacC1*, *aadA1*,^*b*^ *bla*_TEM-1D_, *sul1*	
						Tn*2006*: *oxa23*	
MDR-UNC	2012	USA	450	63	9	AbGRI1-5: *strA*, *strB*, *sul2*, *tetA*(B)	CP031444
						AbGRI2: *aac(6′)-Im*, *aacC1*	
OC043	Unknown	Germany	450	63	9	AbGRI1-3: *strA*, *strB*, *sul2*, *tetA*(B)	CP087321
						AbGRI2: *aac(6′)-Im*, *aacC1*, *aadA1*^*b*^	

a *ampC-19*, *ampC-23*, and *ampC-63* each differ from *ampC-2* by only 2 SNDs.

b Split by an IS*26*-mediated inversion.

**FIG 4:**
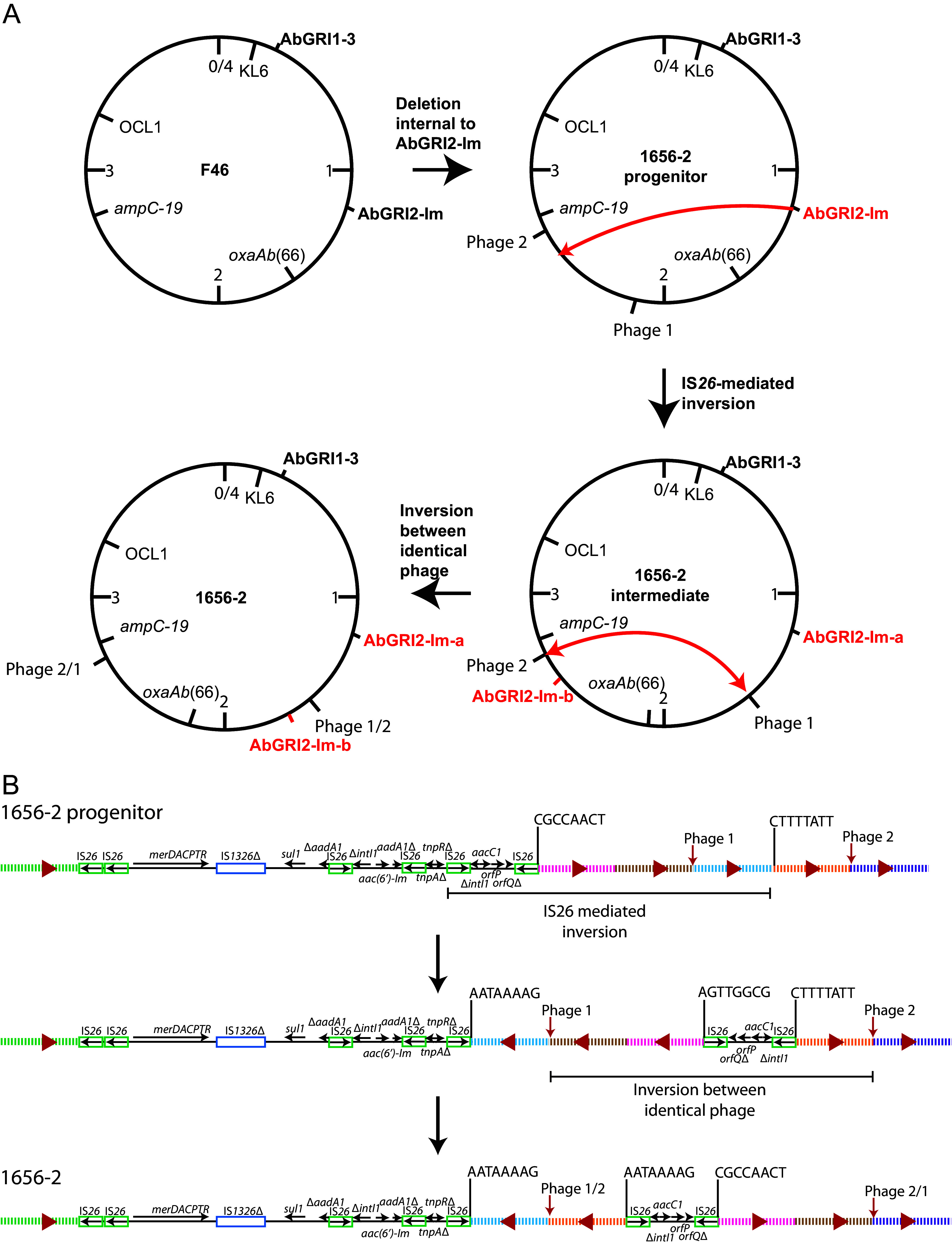
Reconstruction of the original AbGRI2-Im in 1656-2. (A) Consecutive inversions leading to the configuration seen in the complete 1656-2 chromosome. The locations of key features, including AbGRI1, AbGRI2, *oxaAb*, *ampC*, KL, OCL, and two phage, are marked. A red one-headed arrow indicates an IS*26*-mediated inversion originating from AbGRI2, and a red double-headed arrow indicates an inversion occurring between two closely related phage. (B) Detail of inversions leading to the configuration of AbGRI2-Im seen in 1656-2. Labeling underneath lines 1 and 2 indicates the fragments that are inverted in each step. Horizontal arrows show the extend and direction of reading frames. Insertion sequences are shown as boxes. The backbone segments are indicated by different colored lines, with red triangles indicating the relative orientation of each backbone piece. Phage genome positions are marked by vertical red arrows. TSDs are shown as letters above vertical lines. Resistance islands are drawn to scale from GenBank accession number CP001921; backbone spans are not to scale.

Determining the original AbGRI2 configuration in 1656-2 (Fig. 3B) was complicated by two inversions (Fig. 4A). One is an IS*26*-mediated inversion that has split the island, and the other is an inversion between two inversely oriented closely related (>98% nucleotide identity) integrated phage genomes that are not present in the F46 chromosome (Fig. 4B). Reversal of the inversions to give the presumed progenitor showed that it is derived from AbGRI2-Im in F46 (Fig. 3B), differing only by an IS*26*-mediated deletion of 8,084 bp that has removed the span between *tnpR* and the IS*26* adjacent to *intI1* (Fig. 3B). The chromosomal deletion to the left of AbGRI2 in 1656-2 is identical to the 70,280-bp deletion in F46, consistent with a shared origin.

A second complete genome from South Korea, DU202 (GenBank accession number CP017152), which carries *aac*(*6*′)-*Im* contains AbGRI1-3 as in F46 but has also acquired an AbGRI3 island (Table 2). Compared to AbGRI2 in F46, the corresponding island in DU202 differs by a further IS*26*-mediated deletion that has removed 943 bp at the left end of the island and has extended 7,501 bp further into the adjacent chromosome. At the right end of the island, an additional IS*26*-mediated inversion has occurred relative to F46, and an IS*26*-mediated deletion has removed the remaining span of the class 1 integron and 12,002 bp of the adjacent chromosome (Fig. 3B).

The 1656-2 chromosome carries 15 ISAba1 copies, but only the ISAba1 upstream of *ampC* and four more of them at positions 10 to 13 (Fig. 2D) were found in DU202. However, these positions are not shared with any of the Australian ST2:ST423:KL6:OCL1 isolates, indicative of a separate evolutionary pathway from the common ancestor. Relative to F46, 1656-2 and DU202 have 118 and 491 SNDs, respectively, indicative of significant separation.

### *aac*(*6*′)-*Im* found in complete genomes of GC2 isolates with different capsule types.

**FIG 5:**
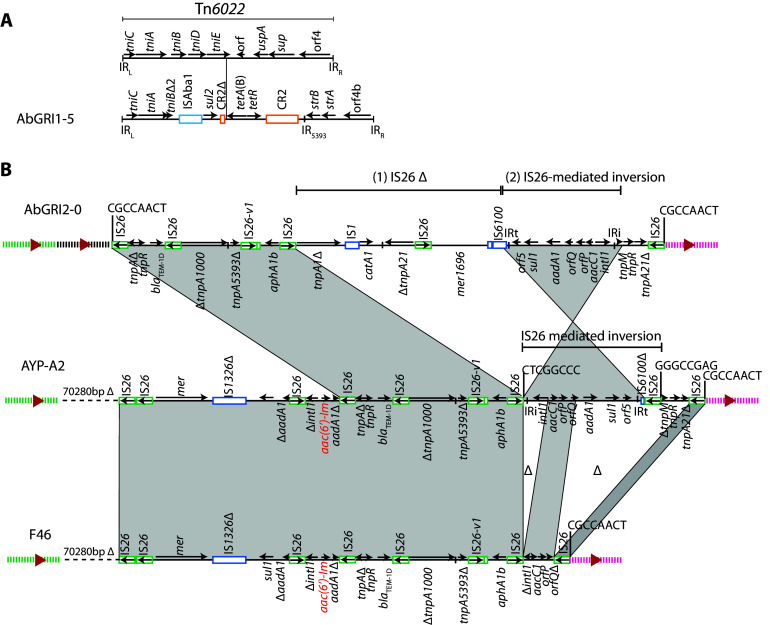
Genomic resistance islands in AYP-A2. (A and B) AbGRI1-5 (A) and AbGRI2-Im variant (B) in AYP-A2 compared to the AbGRI2-0 progenitor and AbGRI2-Im in F46. Horizontal arrows show the extent and direction of reading frames. Insertion sequences are shown as boxes. Shading indicates shared regions, and the backbone adjacent to AbGRI2-Im is indicated by green or pink dashed vertical lines. Dashed horizontal lines indicate backbone deletions, with the size of the deletion shown above. Internal IS*26*-mediated deletions and inversions are marked above or between the lines. TSDs are shown as letters above vertical lines. Illustrations are drawn to scale from GenBank accession numbers CP096575.

In addition to the complete genomes of F46 and the South Korean isolates described above, the *aac*(*6*′)-*Im* gene cassette was found in 13 other complete GC2 A. baumannii genomes (Table 2). These strains were recovered in Australia, the United States, Belgium, Thailand, and Germany between 2004 and 2017. The majority are ST2:ST208:KL2:OCL1 (ST423 is a double-locus variant [DLV] of ST208). The remaining isolates are ST2:ST450:KL9:OCL1, ST2:ST425:KL40:OCL1, ST2:ST448, KL40:OCL1, and ST2:ST1962:KL52:OCL1. Only isolate OC043, from Germany, contains AbGRI1-3 as in F46. Ten of the remaining 12 isolates have AbGRI1-5 (Fig. 5A), which is derived from AbGRI1-3 via homologous recombination (27), and two have AbGRI1-1 (see Tn*6166*, since renamed AbGRI1-1 in reference 41 for structure), which can also be derived from AbGRI1-3 via a different recombination event that removed *sul2*.

The IS*26*-bounded AbGRI2 island of the KL2 (ST208) isolates, including strain AYP-A2, recovered in Victoria, Australia, in 2013 (42), and six isolates, FDAARGOS_560, CFSAN093708, CFSAN093706, CFSAN093707, CFSAN093710, and CFSAN093709, from the United States (2004 to 2007), provides a pathway from the AbGRI2-0 progenitor to the AbGRI2-Im island in F46 (Fig. 5B). In addition to all the fragments found in F46, it also retains a larger span of the class 1 integron in AbGRI2-0, including the *aadA1* and *aacC1* gene cassettes and the *sul1* gene, but this segment has been inverted by IS*26* relative to AbGRI2-0. Reversing this inversion returns AbGRI2 in AYP-A2 to the configuration seen at the right end in AbGRI2-0, with the only difference being an IS*26*-mediated deletion that has removed the span between *tnpA1* and IS*6100* (Fig. 5B). The chromosomal deletion adjacent to the left end of the island in AYP-A2 is identical to the deletion seen in F46, again suggesting that the AbGRI2-Im-type island configurations in these isolates have a common origin. The remaining genomes, CFSAN093705, AB194-VUB, AB3-VUB, CUVET-MIC596, MDR-UNC, and OC043, all possess additional variations of the AbGRI2-Im island in AYP-A2 with larger adjacent chromosomal deletions (data not shown) but retain the *aac*(*6*′)-*Im* gene cassette and novel *mer* regions.

Two alternate routes could link the KL2/ST208 and KL6/ST423 isolates. Either the AbGRI2-Im was shared at some stage between the ST2:ST423:KL6:OCL1 and ST2:ST208:KL2:OCL1 lineages or the K locus in the ancestral AbGRI2-Im strain was replaced by homologous recombination, as is known to occur (8, 40, 43, 44). To examine this possibility, an SND analysis was used to detect recombination patches. Comparison of the AYP-A2 chromosome to that of F46 using Snippy revealed a large (640 kbp) recombination patch spanning across the origin and including both the KL and AbGRI1 (shown as an arc above the circular chromosome in Fig. 2A). This span in AYP-A2 contains 5,572 SNDs relative to the corresponding region in the F46 genome (0.87% difference). In contrast, the remaining ~3.4 Mb of the chromosome contains only 160 SNDs. However, this analysis did not identify which strain is ancestral.

### ISAba1 distribution in complete genomes.

**FIG 6:**
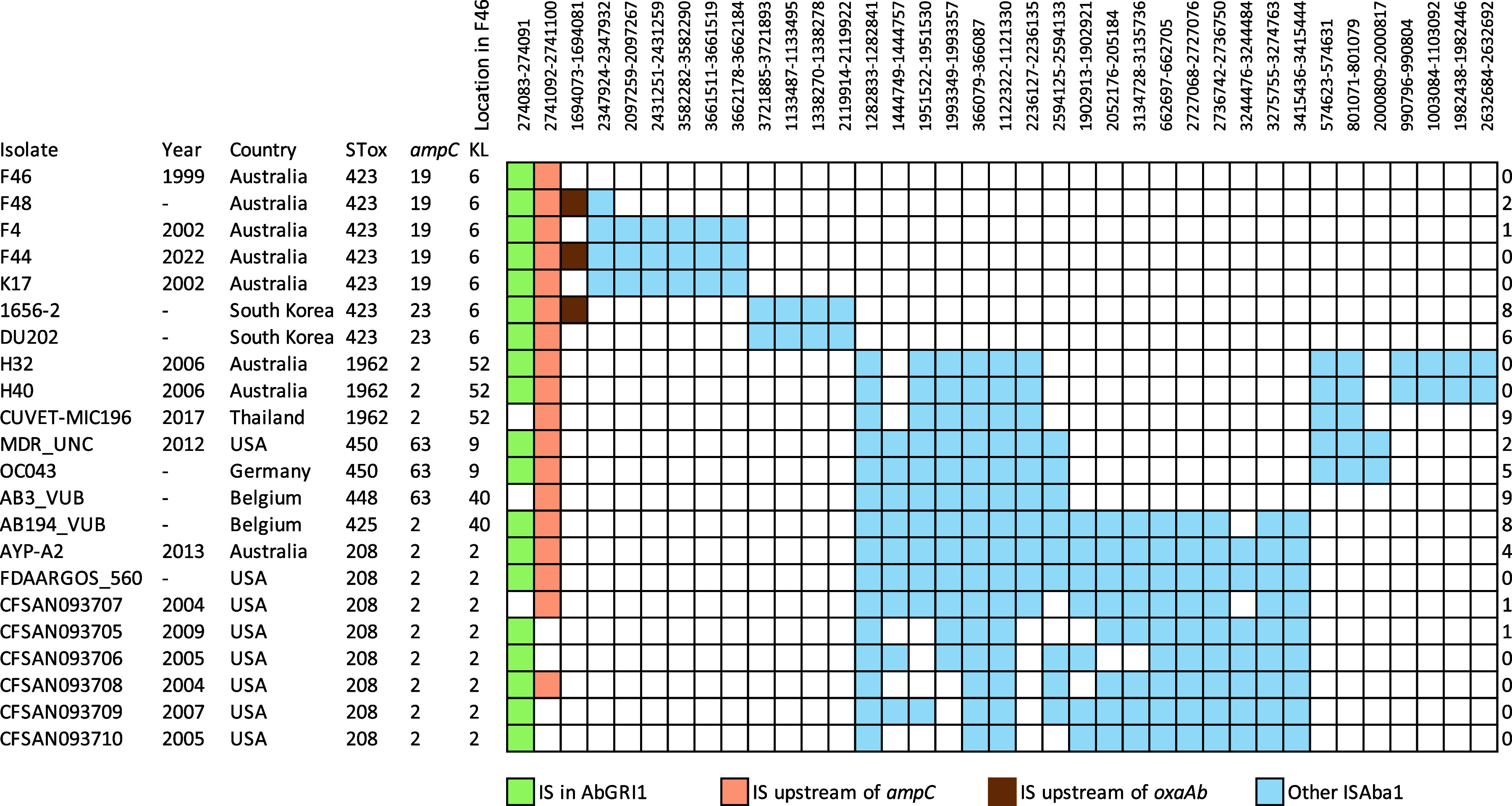
ISAba1 positions shared by groups of A. baumannii genomes containing *aac*(*6*′)-*Im*. Shading, according to the key at the bottom, indicates the presence of a copy of ISAba1 at that location. The locations of numbers 1 to 13 are shown in Fig. 2. The position of the 9-bp target site duplication in the F46 genome (GenBank accession number CP096575) is shown above each IS column. The number at the last column indicates the number of unique copies of ISAba1 in that genome.

One or more events involving movement of ISAba1 to a specific new location are, like single base changes, indicative of shared ancestry. Hence, the ISAba1 positions in the KL2, KL9, KL40, and KL52 isolates were also located. Insertion sequences (IS) shared by two or more genomes are shown in Fig. 6 together with the IS positions in the Australian and South Korean groups. The positions in the F46 genome of the 9-bp duplication surrounding each of these IS are recorded in Fig. 6. Only the ISAba1 in AbGRI1-3 and -5 (AbGRI1-1 lacks this ISAba1) and the copy upstream of *ampC* are shared with either group of KL6 isolates. However, the genomes that include KL2, KL9, KL40, or KL52 all share ISAba1 copies at 8 to 17 additional positions (Fig. 6), consistent with a shared history combined with ongoing ISAba1 spread. Clearly, members of this group have also acquired new KL, presumably via introduction of a recombination patch.

### ST2:ST1962:KL52:OCL1 isolates containing the *aac*(*6*′)-*Im* gene in Australia.

Granted the diversity of KL seen in the complete genomes, we searched our collection of genomes from Australian isolates again using the *aac*(*6*′)-*Im* gene. This revealed that among 41 isolates recovered at the John Hunter Hospital in Newcastle, Australia, 2, H32 and H40 (GenBank accession numbers JASCXA000000000 and JASCXB000000000, respectively), recovered in 2006, carried *aac*(*6*′)-*Im*.

H32 is resistant to amikacin, netilmicin, tobramycin, gentamicin, streptomycin, spectinomycin, ampicillin, ceftazidime, cefotaxime, sulfamethoxazole, tetracycline, trimethoprim, imipenem, meropenem, ciprofloxacin, and nalidixic acid. These resistances were due to the presence of the *aac*(*6*′)-*Im*, *aacC1*, *tet*(B), *strAB*, *sul2*, *bla*_TEM-1b_, and *aadA1* resistance genes in AbGRI1-5 and an AbGRI2-1m variant. In addition, the *oxa23* gene confers resistance to carbapenems. An ISAba1 upstream of the chromosomal *ampC* gene is responsible for resistance to the third generation cephalosporins ceftazidime and cefotaxime, and the appropriate GyrA and ParC substitutions confer nalidixic acid and ciprofloxacin resistance. H40 had a resistance profile and resistance genes similar to those of H32 except for susceptibility to gentamicin due to loss of the *aacC1* gene in H40. H40 also has AbGRI1-5. The AbGRI2-Im islands are deletion derivatives that still retain *aac*(*6*′)-*Im*. In the case of H40, multiple IS*26*-mediated deletions have removed all of the island except for the IS*26*-bounded *aac*(*6*′)-*Im* fragment. However, the characteristic 70,280-bp adjacent deletion was present in both H32 and H40.

Shared ISAba1 positions were identified and are shown in Fig. 6. H32 and H40 cluster with the complete ST2:ST1962:KL52:OCL1 genome, CUVET-MIC596, which was isolated in Thailand in 2017. H32 and H40 also share additional ISAba1 copies (Fig. 6), indicating a close relationship.

### *aac*(*6*′)-*Im* is found in many draft genomes.

A search of the A. baumannii genomes in the GenBank WGS database (1 March 2023) with the *aac*(*6*′)-*Im* gene sequence as a query retrieved a contig from 1,126 draft genomes of isolates that carried this gene (100% identity; 100% coverage). This is a significant proportion (~5%) of the currently available A. baumannii draft genomes. Granted that there are very few published reports of the *aac*(*6*′)-*Im* gene (see Discussion), the number of genomes involved was unexpected and indicates that the presence of this gene is substantially underreported. Indeed, the presence in AYP-A2 was not reported previously (42). These isolates were recovered from 28 different countries over the time span 1999 to 2023 (Table 3). The countries were mainly in Europe, the Middle East, Asia, and North America, but Australia (the isolates reported here and an additional Brisbane isolate from 2003) and Russia are also represented. This indicates a global dissemination. The sequence type of one representative genome from each country was examined, and all were ST2.

**TABLE 3:** Genomes in WGS containing *aac(6′)-Im* (last searched 1 March 2023)

Country	No. of sequences	Yr(s) of isolation
USA	916	2005–2023
Unknown	38	2011–2019
Poland	24	2010–2013
Germany	21	2008–2014
Saudi Arabia	17	2013–2019
Israel	14	2013–2019
Kuwait	12	2015
Spain	10	2010–2011
Serbia	9	2018
Italy	7	2013–2016
Thailand	7	1998–2019
France	6	2014–2019
Russia	6	2014–2019
Australia^*a*^	5	2002–2003
Croatia	4	2018
Nepal	4	2014
Romania	3	2018–2019
South Korea	3	2009–2011
Switzerland	3	2010–2015
Canada	2	2011
Denmark	2	2010
Hungary	2	2012
Japan	2	2015
Kosovo	2	2017–2017
Libya	2	2019
Myanmar	2	2016
Belgium	1	2017
China	1	2013
Sweden	1	2012
Total	1,126	1998–2023

a Includes F4, F44, F48, and K17. The fifth isolate (2003) is also from the Brisbane outbreak and differs by 5 SNDs from F48. H32 and H40 are not included, owing to the search date.

## DISCUSSION

Following the original 1997 description of the *aac*(*6*′)-*Im* in a class 1 integron-associated gene cassette in C. freundii (16) and its discovery in A. baumannii in South Korea in 2008 (15), it was rarely reported. It was found in a single GC2 A. baumannii isolate, said to be imported from Thailand, in a study of sporadic isolates recovered in Sweden (45) and also in a single GC2 A. baumannii isolate recovered between 2013 and 2014 in Nepal (46). The gene was added to the ResFinder database only in 2018 but as *aac*(*6*′)-*Ip* and has been reported recently as *-Ip* in a small number of GC2 isolates from Israel and Italy (47). Granted the absence of a significant number of reports, our finding that *aac*(*6*′)-*Im* is present in 15 complete and over 1,000 draft A. baumannii genomes, representing over 5% of currently available genomes, is surprising. This may reflect the fact that the focus of genomic studies is most often only on resistance to carbapenem antibiotics. However, the modest number of cases in which genomes have been analyzed in detail to document all of the resistance genes present and even fewer cases that assign them to specific known resistance island types is likely another factor. In addition, the *aac*(*6*′)-*Im* gene appears to be almost exclusively present in A. baumannii and likely only in GC2 isolates, as searches of the GenBank nonredundant nucleotide database identified only one case of the gene in another species, namely, the original report (16).

The source of the IS*26*-bounded AbGRI2 island has been identified (48). The proposed original configuration, AbGRI2-0, is present in A320 (RUH134), one of the earliest GC2 isolates for which genome data are available (29) (GenBank accession number CP032055). AbGRI2-0 includes further copies of IS*26*, and as a consequence of their action causing inversions and deletions, a large number of variants of this resistance island have been reported (3, 29), and in many cases, IS*26*-mediated deletion of adjacent DNA is also observed. An inversion of the chromosome splitting AbGRI2, as seen in this study for 1656-2, is also seen in the A320 genome (29). However, the configurations reported here, which for simplicity and clarity we have designated collectively as AbGRI2-Im variants, are all derived from the configuration that arose as a consequence of the initial incorporation of an additional IS*26*-bounded segment. This AbGRI2 type had not been described previously. The addition also appears to have resulted from the action of IS*26* in the targeted conservative mode (49, 50) or via homologous recombination within the boundaries of IS*26*. Either prior or subsequent to this acquisition event, an IS*26*-mediated deletion of adjacent chromosomal DNA with a characteristic length occurred. Evolution of AbGRI2-Im *in situ* has continued to be shaped by the action of IS*26*, as evidenced by the several variations reported here (Fig. 1, 3, and 5).

Based on the analysis of ISAba1 distribution in the isolates described or analyzed in this study, the 1999 isolate F46 we sequenced appears to be close to the progenitor of all of the groups with different sets of shared ISAba1 locations. Indeed, it seems likely that ISAba1 entered the GC2 forebear in AbGRI1, as this IS is in the transposon that is the source of the ancestor of the AbGRI1-type resistance islands (28). If this is correct, an ISAba1 subsequently moved to the position upstream of *ampC*. This would parallel the situation in global clone 1, where it is known that the earliest isolates do not include any ISAba1 copies (51; unpublished observations). In one GC1 sublineage, the source of the ISAba1 has been traced to the entry of Tn*6168* carrying a second copy of the *ampC* gene, and two further sublineages could be distinguished via the different patterns of shared ISAba1 positions (43). Further work tracking ISAba1 copies in genomes of isolates from different continents and countries may shed light on how this GC2 type has spread. For, example, the 1656-2 genome, which carries an AbGRI2-Im variant, was included in a phylogeny of GC2 isolates (52), and this revealed a close relationship with two isolates from an earlier study of a polyclonal outbreak in 2007 at the NIH Clinical Center, Bethesda, MD (44). Resistance genes were not examined in that study, but our examination of these draft genomes revealed that both isolates carried *aac*(*6*′)-*Im.* The authors reported recombinational exchange involving the K locus, but the study predates the assignment of KL numbers (40). We found that one isolate, designated NIH-3, carried KL6 while the other, NIH-2, carried KL9. Analysis of the ISAba1 locations in the KL6 isolate relative to those found in the Australian and South Korean KL6 groups may provide insight into the source of this isolate.

It is well known that extensive recombinational exchange occurs in A. baumannii, and this complicates analysis of the relationships within clonal complexes. Among the earliest examples were exchanges of the region that includes that capsule locus (KL) (40), and the isolates that include AbGRI2-Im with different KL found in this study could have arisen by import of a different KL-containing segment. Recombination in this region was observed in the comparison of the F46 (KL6) and AYP-A2 (KL2) genomes and was reported previously for the NIH-3 (KL6) and NIH-2 (KL9) strains described above (44). Alternatively, import of the AbGRI2-Im region could also have occurred. Further work in the form of an in-depth analysis (ISAba1 distribution, SND analysis to detect large recombination patches, etc.) will be needed to determine if these two possibilities can be distinguished.

Finally, in the course of this study we detected a serious problem with the names assigned to aminoglycoside resistance genes, as two quite distinct genes are listed as *aac*(*6*′)-*Im*. The gene described here is the first reported and hence retains the designation *-Im*. The second was published several years later (53) and by convention must be renamed. We suggest *-In*, as this designation is currently not assigned.

## MATERIALS AND METHODS

### Bacterial strains.

Acinetobacter baumannii isolates used are listed in Table 1. F4, F44, F46, and F48 (kindly supplied by Mohammad Katouli) are part of the collection of isolates reported previously (22) that were collected between 1999 and 2002 at the Royal Brisbane and Women’s Hospital (Brisbane, Australia). These isolates are also referred to as RBH4, RBH44, RBH46, and RBH48. Isolate K17 (original name isolate 6856390) was collected in 2002 at the Royal Adelaide Hospital (32) and was kindly supplied by Melissa Brown. Isolates H32 and H40 were collected at the John Hunter Hospital (Newcastle, Australia) in 2006 and were kindly supplied by John Ferguson.

### PCR.

The *aac*(*6*′)-*Im* gene was amplified from whole-cell DNA using primers RH569 and RH570 (14) under standard conditions (60°C annealing temperature, 30-s extension time, 30 cycles) using *Taq* DNA polymerase (New England Biolabs) according to manufacturer’s instructions. The PCR amplicon was sequenced by Sanger sequencing (Australian Genome Research Facility) to confirm the identity of the amplicon. PCR, carried out using standard conditions described above, was used to establish linkages between contigs in the draft genomes associated with resistance genes that were detected using Abricate version 1.0.0. Primers were described previously (29) or designed based on the F46 sequence to span the region between additional pairs of contigs.

### Genome sequencing and assembly.

Whole-cell DNA was prepared and sequenced on an Illumina HiSeq platform at the Wellcome Trust Sanger Institute (Cambridge, UK) as described previously (8). Sequencing generated 1,500,338 to 2,645,824 paired-end reads that were 100 bp in length after trimming to remove adapters. This represents 42- to 66-fold coverage of the entire genome (Table S1). Read quality was validated using FastQC (https://qubeshub.org/resources/fastqc). For draft genomes, the Illumina reads were assembled *de novo* using SPAdes (version 3.15.5) with default assembly parameters (k-mers of 21, 33, 55, and 77). Assemblies were checked for completeness and contamination using CheckM (54).

The antibiotic resistance islands were assembled from contigs in the draft using linkage PCR as described above, to link adjacent contigs generating enhanced draft assemblies containing 66 to 109 contigs (Table S1).

Whole-cell DNA from one isolate, F46, was also sequenced on a PacBio RS platform (DNA Link, South Korea) as described previously (35) to resolve complex repeat regions. This generated 182,344 reads with an average length of 6,028 bp, representing 283-fold coverage. The Illumina and PacBio reads from F46 were assembled *de novo* in a hybrid assembly using Unicycler version 0.4.0 (55) with default assembly parameters as described previously (35).

The protein-coding, rRNA, and tRNA gene sequences were annotated using Prokka version 1.14.5, the polysaccharide loci were identified and annotated using Kaptive version 2.0.0 (56), and the antibiotic resistance genes were annotated manually to correct names assigned by Abricate. Multilocus sequence typing (MLST) was done *in silico* using PubMLST (http://pubmlst.org). The *ampC* allele was identified using the *ampC* database on the PubMLST platform (57).

### Antibiotic resistance and analysis of resistance regions.

Resistance to antibiotics was determined using a standard disk diffusion assay. Meropenem MICs were determined by plating on doubling concentrations of meropenem or with meropenem Etest strips (bioMérieux, France) with a known meropenem-sensitive isolate, G13 (33), as a control. Antibiotic resistance regions in complete and draft genomes were identified as AbGRI1, AbGRI2, or AbGRI3 by their location and their content was typed using an in-house database of known AbGRI1, AbGRI2, and AbGRI3 structures (11, 27, 58) with stand-alone BLAST.

### Identification of genomes carrying *aac*(*6*′)-*Im*.

Genomes containing *aac*(*6*′)-*Im* were identified by performing a BLASTn search (https://blast.ncbi.nlm.nih.gov/Blast.cgi) of the GenBank nonredundant nucleotide or whole-genome sequence (WGS) databases with the *aac*(*6*′)-*Im* gene as the query. Cutoffs of 99% identity and 100% coverage were applied. The original *aac*(*6*′)-*Im* gene sequence (GenBank accession number Z54241) was found to contain a single nucleotide deletion that introduces a frameshift near the C terminus. Hence, the *aac*(*6*′)-*Im* (but annotated as *aacA16* [19]) gene sequence (GenBank accession number NG052380) which is derived from the complete genome of A. baumannii strain 1656-2 (GenBank accession number CP001921) was used as the query.

### ISAba1 mapping.

ISMapper (59) was used to map the locations of copies of ISAba1 in strains examined in this study. The complete genome of F46, the oldest isolate in the collection, was used as the reference sequence. The sequence of ISAba1, retrieved from ISFinder (https://isfinder.biotoul.fr/), was used as the query sequence, and paired-end Illumina reads were the input for the remaining isolates of interest. Where paired-end reads were not available for completed genomes in the public domain, one million MiSeq reads were simulated from the FASTA file of the complete genome using InSilicoSeq version 1.5.4 (60). After mapping, the individual output text files were compiled using a compiled_table.py script and were sorted by prevalence. The individual IS locations in the chromosome were compiled and identified by their location in the F46 reference genome.

### Mapping of SNDs.

Single nucleotide differences (SNDs) were mapped using Snippy version 3.0 (https://github.com/tseemann/snippy) with default parameters on the University of Sydney Artemis High Performance Computing cluster. The complete F46 genome was used as the reference sequence, with paired-end Illumina reads from F4, F44, F48, K17, and AYP-A2 as a query input. Illumina reads for the South Korean isolates 1656-2 and DU202 were not available, so their complete genomes were used as a reference with the F46 paired-end Illumina reads as a query. For comparisons between F4, F44, F48, and K17, the reads of each isolate and the draft sequence of each isolate were used sequentially as either the query or the reference.

### Data availability.

The draft sequences of F4, F4, F48, K17, H32, and H40 and the complete genome of F46 have been deposited in GenBank (BioProject PRJNA812061) under accession numbers JAKZLG000000000, JAQJIR000000000, JAQJIQ000000000, JAQJIP000000000, JASCXA000000000, JASCXB000000000, and CP096575, respectively. Reads for all samples are available under the BioSample and SRA accession numbers listed in the respective GenBank accessions.

## References

[1] Nguyen M, Joshi SG. 2021. Carbapenem resistance in *Acinetobacter baumannii*, and their importance in hospital-acquired infections: a scientific review. J Appl Microbiol 131:2715–2738. doi:10.1111/jam.15130. 33971055

[2] Becker B, Cooper MA. 2013. Aminoglycoside antibiotics in the 21st century. ACS Chem Biol 8:105–115. doi:10.1021/cb3005116. 23110460

[3] Nigro SJ, Hall RM. 2016. Loss and gain of aminoglycoside resistance in global clone 2 *Acinetobacter baumannii* in Australia via modification of genomic resistance islands and acquisition of plasmids. J Antimicrob Chemother 71:2432–2440. doi:10.1093/jac/dkw176. 27246238

[4] Peleg AY, Seifert H, Paterson DL. 2008. *Acinetobacter baumannii*: emergence of a successful pathogen. Clin Microbiol Rev 21:538–582. doi:10.1128/CMR.00058-07. 18625687 PMC2493088

[5] Seward RJ, Lambert T, Towner KJ. 1998. Molecular epidemiology of aminoglycoside resistance in *Acinetobacter* spp. J Med Microbiol 47:455–462. doi:10.1099/00222615-47-5-455. 9879947

[6] McGann P, Courvalin P, Snesrud E, Clifford RJ, Yoon EJ, Onmus-Leone F, Ong AC, Kwak YI, Grillot-Courvalin C, Lesho E, Waterman PE. 2014. Amplification of aminoglycoside resistance gene aphA1 in *Acinetobacter baumannii* results in tobramycin therapy failure. mBio 5:e00915-14. doi:10.1128/mBio.00915-14. PMC3994513 24757213

[7] Doi Y, Wachino J-I, Arakawa Y. 2016. Aminoglycoside resistance: the emergence of acquired 16S ribosomal RNA methyltransferases. Infect Dis Clin North Am 30:523–537. doi:10.1016/j.idc.2016.02.011. 27208771 PMC4878400

[8] Holt K, Kenyon JJ, Hamidian M, Schultz MB, Pickard DJ, Dougan G, Hall R. 2016. Five decades of genome evolution in the globally distributed, extensively antibiotic-resistant *Acinetobacter baumannii* global clone 1. Microb Genom 2:e000052. doi:10.1099/mgen.0.000052. 28348844 PMC5320584

[9] Hamidian M, Hall RM. 2018. The AbaR antibiotic resistance islands found in *Acinetobacter baumannii* global clone 1—structure, origin and evolution. Drug Resist Updat 41:26–39. doi:10.1016/j.drup.2018.10.003. 30472242

[10] Harmer CJ, Lebreton F, Stam J, McGann PT, Hall RM. 2022. Mechanisms of IS26-mediated amplification of the aphA1 gene leading to tobramycin resistance in an *Acinetobacter baumannii* isolate. Microbiol Spectr 10:e02287-22. doi:10.1128/spectrum.02287-22. PMC9602291 36073931

[11] Blackwell GA, Holt KE, Bentley SD, Hsu LY, Hall RM. 2017. Variants of AbGRI3 carrying the armA gene in extensively antibiotic-resistant *Acinetobacter baumannii* from Singapore. J Antimicrob Chemother 72:1031–1039. doi:10.1093/jac/dkw542. 28073968 PMC5400096

[12] Doi Y, Arakawa Y. 2007. 16S ribosomal RNA methylation: emerging resistance mechanism against aminoglycosides. Clin Infect Dis 45:88–94. doi:10.1086/518605. 17554708

[13] Hamidian M, Nigro SJ, Hall RM. 2012. Variants of the gentamicin and tobramycin resistance plasmid pRAY are widely distributed in *Acinetobacter*. J Antimicrob Chemother 67:2833–2836. doi:10.1093/jac/dks318. 22888272

[14] Nigro SJ, Post V, Hall RM. 2011. Aminoglycoside resistance in multiply antibiotic-resistant *Acinetobacter baumannii* belonging to global clone 2 from Australian hospitals. J Antimicrob Chemother 66:1504–1509. doi:10.1093/jac/dkr163. 21586593

[15] Han HL, Jang SJ, Park G, Kook JK, Shin JH, Shin SH, Kim DM, Cheon JS, Moon DS, Park YJ. 2008. Identification of an atypical integron carrying an IS26-disrupted aadA1 gene cassette in *Acinetobacter baumannii*. Int J Antimicrob Agents 32:165–169. doi:10.1016/j.ijantimicag.2008.03.009. 18565738

[16] Hannecart-Pokorni E, Depuydt F, de Wit L, van Bossuyt E, Content J, Vanhoof R. 1997. Characterization of the 6′-N-aminoglycoside acetyltransferase gene aac(6′)-Im [corrected] associated with a sulI-type integron. Antimicrob Agents Chemother 41:314–318. doi:10.1128/AAC.41.2.314. 9021185 PMC163707

[17] Vanhoof R, Hannecart-Pokorni E, Content J. 1998. Nomenclature of genes encoding aminoglycoside-modifying enzymes. Antimicrob Agents Chemother 42:483. doi:10.1128/AAC.42.2.483. 9527817 PMC105445

[18] Centron D, Roy PH. 1998. Characterization of the 6′-N-aminoglycoside acetyltransferase gene aac(6′)-Iq from the integron of a natural multiresistance plasmid. Antimicrob Agents Chemother 42:1506–1508. doi:10.1128/AAC.42.6.1506. 9624504 PMC105632

[19] Partridge SR, Tsafnat G, Coiera E, Iredell JR. 2009. Gene cassettes and cassette arrays in mobile resistance integrons. FEMS Microbiol Rev 33:757–784. doi:10.1111/j.1574-6976.2009.00175.x. 19416365

[20] Runnegar N, Sidjabat H, Goh HM, Nimmo GR, Schembri MA, Paterson DL. 2010. Molecular epidemiology of multidrug-resistant *Acinetobacter baumannii* in a single institution over a 10-year period. J Clin Microbiol 48:4051–4056. doi:10.1128/JCM.01208-10. 20739495 PMC3020849

[21] Hamidian M, Nigro SJ, Hall RM. 2017. Problems with the Oxford multilocus sequence typing scheme for *Acinetobacter baumannii*: do sequence type 92 (ST92) and ST109 exist? J Clin Microbiol 55:2287–2289. doi:10.1128/JCM.00533-17. 28490493 PMC5483935

[22] Long YB, Faoagali J, Bodman J, George N, McKay D, Katouli M. 2009. Persistence of multiple antibiotic resistant strains of *Acinetobacter baumannii* carrying class 1 integron in a hospital setting. Microb Drug Resist 15:167–172. doi:10.1089/mdr.2009.0884. 19728773

[23] Kenyon JJ, Marzaioli AM, Hall RM, De Castro C. 2015. Structure of the K6 capsular polysaccharide from *Acinetobacter baumannii* isolate RBH4. Carbohydr Res 409:30–35. doi:10.1016/j.carres.2015.03.016. 25917131

[24] Hamidian M, Ambrose SJ, Blackwell GA, Nigro SJ, Hall RM. 2021. An outbreak of multiply antibiotic-resistant ST49:ST128:KL11:OCL8 *Acinetobacter baumannii* isolates at a Sydney hospital. J Antimicrob Chemother 76:893–900. doi:10.1093/jac/dkaa553. 33452522

[25] Hamidian M, Hall RM. 2013. ISAba1 targets a specific position upstream of the intrinsic ampC gene of *Acinetobacter baumannii* leading to cephalosporin resistance. J Antimicrob Chemother 68:2682–2683. doi:10.1093/jac/dkt233. 23788477

[26] Huang H, Yang ZL, Wu XM, Wang Y, Liu YJ, Luo H, Lv X, Gan YR, Song SD, Gao F. 2012. Complete genome sequence of *Acinetobacter baumannii* MDR-TJ and insights into its mechanism of antibiotic resistance. J Antimicrob Chemother 67:2825–2832. doi:10.1093/jac/dks327. 22952140

[27] Nigro SJ, Brown MH, Hall RM. 2019. AbGRI1-5, a novel AbGRI1 variant in an *Acinetobacter baumannii* GC2 isolate from Adelaide, Australia. J Antimicrob Chemother 74:821–823. doi:10.1093/jac/dky459. 30452642

[28] Hamidian M, Hall RM. 2017. Origin of the AbGRI1 antibiotic resistance island found in the comM gene of *Acinetobacter baumannii* GC2 isolates. J Antimicrob Chemother 72:2944–2947. doi:10.1093/jac/dkx206. 28666372

[29] Blackwell GA, Nigro SJ, Hall RM. 2015. Evolution of AbGRI2-0, the progenitor of the AbGRI2 resistance island in global clone 2 of *Acinetobacter baumannii*. Antimicrob Agents Chemother 60:1421–1429. doi:10.1128/AAC.02662-15. 26666934 PMC4775934

[30] Partridge SR, Hall RM. 2004. Complex multiple antibiotic and mercury resistance region derived from the r-det of NR1 (R100). Antimicrob Agents Chemother 48:4250–4255. doi:10.1128/AAC.48.11.4250-4255.2004. 15504849 PMC525457

[31] Partridge SR. 2011. Analysis of antibiotic resistance regions in Gram-negative bacteria. FEMS Microbiol Rev 35:820–855. doi:10.1111/j.1574-6976.2011.00277.x. 21564142

[32] Eijkelkamp BA, Stroeher UH, Hassan KA, Papadimitrious MS, Paulsen IT, Brown MH. 2011. Adherence and motility characteristics of clinical *Acinetobacter baumannii* isolates. FEMS Microbiol Lett 323:44–51. doi:10.1111/j.1574-6968.2011.02362.x. 22092679

[33] Nigro SJ, Hall RM. 2018. Does the intrinsic oxaAb (blaOXA-51-like) gene of *Acinetobacter baumannii* confer resistance to carbapenems when activated by ISAba1? J Antimicrob Chemother 73:3518–3520. doi:10.1093/jac/dky334. 30124881

[34] Takebayashi Y, Findlay J, Heesom KJ, Warburton PJ, Avison MB, Evans BA. 2021. Variability in carbapenemase activity of intrinsic OxaAb (OXA-51-like) beta-lactamase enzymes in *Acinetobacter baumannii*. J Antimicrob Chemother 76:587–595. doi:10.1093/jac/dkaa502. 33338207

[35] Nigro SJ, Wick R, Holt KE, Hall RM. 2018. Complete genome sequence of WM99c, an antibiotic-resistant *Acinetobacter baumannii* global clone 2 (GC2) strain representing an Australian GC2 lineage. Microbiol Resour Announc 7:e01199-18. doi:10.1128/MRA.01199-18. 30533856 PMC6284088

[36] Adams MD, Bishop B, Wright MS. 2016. Quantitative assessment of insertion sequence impact on bacterial genome architecture. Microb Genom 2:e000062. doi:10.1099/mgen.0.000062. 28348858 PMC5343135

[37] Feng Y, Ruan Z, Shu J, Chen CL, Chiu CH. 2016. A glimpse into evolution and dissemination of multidrug-resistant *Acinetobacter baumannii* isolates in East Asia: a comparative genomics study. Sci Rep 6:24342. doi:10.1038/srep24342. 27072398 PMC4829828

[38] Park JY, Kim S, Kim SM, Cha SH, Lim SK, Kim J. 2011. Complete genome sequence of multidrug-resistant *Acinetobacter baumannii* strain 1656-2, which forms sturdy biofilm. J Bacteriol 193:6393–6394. doi:10.1128/JB.06109-11. 22038960 PMC3209198

[39] Lee SY, Yun SH, Lee YG, Choi CW, Leem SH, Park EC, Kim GH, Lee JC, Kim SI. 2014. Proteogenomic characterization of antimicrobial resistance in extensively drug-resistant *Acinetobacter baumannii* DU202. J Antimicrob Chemother 69:1483–1491. doi:10.1093/jac/dku008. 24486871

[40] Kenyon JJ, Hall RM. 2013. Variation in the complex carbohydrate biosynthesis loci of *Acinetobacter baumannii* genomes. PLoS One 8:e62160. doi:10.1371/journal.pone.0062160. 23614028 PMC3628348

[41] Nigro SJ, Hall RM. 2012. Antibiotic resistance islands in A320 (RUH134), the reference strain for *Acinetobacter baumannii* global clone 2. J Antimicrob Chemother 67:335–338. doi:10.1093/jac/dkr447. 22020138

[42] Hawkey J, Ascher DB, Judd LM, Wick RR, Kostoulias X, Cleland H, Spelman DW, Padiglione A, Peleg AY, Holt KE. 2018. Evolution of carbapenem resistance in *Acinetobacter baumannii* during a prolonged infection. Microb Genom 4:e000165. doi:10.1099/mgen.0.000165. 29547094 PMC5885017

[43] Hamidian M, Hawkey J, Wick R, Holt KE, Hall RM. 2019. Evolution of a clade of *Acinetobacter baumannii* global clone 1, lineage 1 via acquisition of carbapenem- and aminoglycoside-resistance genes and dispersion of ISAba1. Microb Genom 5:e000242. doi:10.1099/mgen.0.000242. 30648939 PMC6412058

[44] Snitkin ES, Zelazny AM, Montero CI, Stock F, Mijares L, Program NCS, Murray PR, Segre JA, NISC Comparative Sequence Program. 2011. Genome-wide recombination drives diversification of epidemic strains of *Acinetobacter baumannii*. Proc Natl Acad Sci USA 108:13758–13763. doi:10.1073/pnas.1104404108. 21825119 PMC3158218

[45] Karah N, Haldorsen B, Hermansen NO, Tveten Y, Ragnhildstveit E, Skutlaberg DH, Tofteland S, Sundsfjord A, Samuelsen O. 2011. Emergence of OXA-carbapenemase- and 16S rRNA methylase-producing international clones of *Acinetobacter baumannii* in Norway. J Med Microbiol 60:515–521. doi:10.1099/jmm.0.028340-0. 21163830

[46] Shrestha S, Tada T, Miyoshi-Akiyama T, Ohara H, Shimada K, Satou K, Teruya K, Nakano K, Shiroma A, Sherchand JB, Rijal BP, Hirano T, Kirikae T, Pokhrel BM. 2015. Molecular epidemiology of multidrug-resistant *Acinetobacter baumannii* isolates in a university hospital in Nepal reveals the emergence of a novel epidemic clonal lineage. Int J Antimicrob Agents 46:526–531. doi:10.1016/j.ijantimicag.2015.07.012. 26362951

[47] Frenk S, Temkin E, Lurie-Weinberger MN, Keren-Paz A, Rov R, Rakovitsky N, Wullfhart L, Nutman A, Daikos GL, Skiada A, Durante-Mangoni E, Dishon Benattar Y, Bitterman R, Yahav D, Daitch V, Bernardo M, Iossa D, Zusman O, Friberg LE, Mouton JW, Theuretzbacher U, Leibovici L, Geffen Y, Gershon R, Paul M, Carmeli Y. 2022. Large-scale WGS of carbapenem-resistant *Acinetobacter baumannii* isolates reveals patterns of dissemination of ST clades associated with antibiotic resistance. J Antimicrob Chemother 77:934–943. doi:10.1093/jac/dkac010. 35084023

[48] Blackwell GA, Hamidian M, Hall RM. 2016. IncM plasmid R1215 is the source of chromosomally located regions containing multiple antibiotic resistance genes in the globally disseminated *Acinetobacter baumannii* GC1 and GC2 clones. mSphere 1:e00117-16. doi:10.1128/mSphere.00117-16. 27303751 PMC4899885

[49] Harmer CJ, Moran RA, Hall RM. 2014. Movement of IS26-associated antibiotic resistance genes occurs via a translocatable unit that includes a single IS26 and preferentially inserts adjacent to another IS26. mBio 5:e01801-14. doi:10.1128/mBio.01801-14. 25293759 PMC4196232

[50] Harmer CJ, Hall RM. 2016. IS26-mediated formation of transposons carrying antibiotic resistance genes. mSphere 1:e00038-16. doi:10.1128/mSphere.00038-16. PMC4894685 27303727

[51] Holt KE, Hamidian M, Kenyon JJ, Wynn MT, Hawkey J, Pickard D, Hall RM. 2015. Genome sequence of *Acinetobacter baumannii* strain A1, an early example of antibiotic-resistant global clone 1. Genome Announc 3:e00032-15. doi:10.1128/genomeA.00032-15. 25767221 PMC4357743

[52] Wright MS, Iovleva A, Jacobs MR, Bonomo RA, Adams MD. 2016. Genome dynamics of multidrug-resistant *Acinetobacter baumannii* during infection and treatment. Genome Med 8:26. doi:10.1186/s13073-016-0279-y. 26939581 PMC4776386

[53] Chow JW, Kak V, You I, Kao SJ, Petrin J, Clewell DB, Lerner SA, Miller GH, Shaw KJ. 2001. Aminoglycoside resistance genes aph(2″)-Ib and aac(6′)-Im detected together in strains of both Escherichia coli and Enterococcus faecium. Antimicrob Agents Chemother 45:2691–2694. doi:10.1128/AAC.45.10.2691-2694.2001. 11557456 PMC90718

[54] Parks DH, Imelfort M, Skennerton CT, Hugenholtz P, Tyson GW. 2015. CheckM: assessing the quality of microbial genomes recovered from isolates, single cells, and metagenomes. Genome Res 25:1043–1055. doi:10.1101/gr.186072.114. 25977477 PMC4484387

[55] Wick RR, Judd LM, Gorrie CL, Holt KE. 2017. Unicycler: resolving bacterial genome assemblies from short and long sequencing reads. PLoS Comput Biol 13:e1005595. doi:10.1371/journal.pcbi.1005595. 28594827 PMC5481147

[56] Wyres KL, Cahill SM, Holt KE, Hall RM, Kenyon JJ. 2020. Identification of *Acinetobacter baumannii* loci for capsular polysaccharide (KL) and lipooligosaccharide outer core (OCL) synthesis in genome assemblies using curated reference databases compatible with Kaptive. Microb Genom 6:e000339. doi:10.1099/mgen.0.000339. 32118530 PMC7200062

[57] Karah N, Jolley KA, Hall RM, Uhlin BE. 2017. Database for the ampC alleles in *Acinetobacter baumannii*. PLoS One 12:e0176695. doi:10.1371/journal.pone.0176695. 28459877 PMC5411055

[58] Nigro SJ, Farrugia DN, Paulsen IT, Hall RM. 2013. A novel family of genomic resistance islands, AbGRI2, contributing to aminoglycoside resistance in *Acinetobacter baumannii* isolates belonging to global clone 2. J Antimicrob Chemother 68:554–557. doi:10.1093/jac/dks459. 23169892

[59] Hawkey J, Hamidian M, Wick RR, Edwards DJ, Billman-Jacobe H, Hall RM, Holt KE. 2015. ISMapper: identifying transposase insertion sites in bacterial genomes from short read sequence data. BMC Genomics 16:667. doi:10.1186/s12864-015-1860-2. 26336060 PMC4558774

[60] Gourle H, Karlsson-Lindsjo O, Hayer J, Bongcam-Rudloff E. 2019. Simulating Illumina metagenomic data with InSilicoSeq. Bioinformatics 35:521–522. doi:10.1093/bioinformatics/bty630. 30016412 PMC6361232

