# Supplementary material for: Acinetobacter baumannii GC2 Sublineage Carrying the aac(6′)-Im Amikacin, Netilmicin, and Tobramycin Resistance Gene Cassette
Source: Microbiol Spectr. 2023 Jul 6;11(4):e01204-23. doi: 10.1128/spectrum.01204-23 (PMC10434200; doi:10.1128/spectrum.01204-23)
Supplement: Supplemental file 1 — Table S1. Download spectrum.01204-23-s0001.docx, DOCX file, 0.01 MB [file spectrum.01204-23-s0001.docx]

Supplementary Table 1 Sequencing and assembly

| Isolate | Number of reads | Read depth | Number of contigs | Average contig length | N50 |
| --- | --- | --- | --- | --- | --- |
| F46 (PacBio) | 182344 | 283 | 1^a^ | 3878974^a^ | N/A |
| F46 (Ilumina) | 1791327 | 46 | N/A | N/A | N/A |
| F4 | 2645824 | 66 | 100^b^ | 35406^b^ | 171031 |
| F44 | 1669656 | 42 | 81^b^ | 42859^b^ | 142553 |
| F48 | 2508104 | 63 | 66^b^ | 50179^b^ | 210401 |
| K17 | 1681610 | 43 | 76^b^ | 39484^b^ | 171165 |
| H32 | 1500338 | 38 | 109^b^ | 35432^b^ | 120693 |
| H40 | 1891041 | 50 | 91^b^ | 42340^b^ | 120709 |

a Hybrid assembly

b Short read only assembly
